# Recurrent Syncope Related to Carotid Compression in Eagle Syndrome: A Case Report

**DOI:** 10.7759/cureus.45134

**Published:** 2023-09-12

**Authors:** Diana Breda, Sandra Ferreira, Margarida Colino, Érica Cerqueira, Isabel Amado

**Affiliations:** 1 Maxillofacial Surgery Department, Centro Hospitalar e Universitário de Coimbra, Coimbra, PRT

**Keywords:** styloidectomy, syncope, elongated styloid syndrome, stylocarotid syndrome, eagle syndrome

## Abstract

Eagle syndrome is a rare disease characterised by symptoms associated with an elongated styloid process or calcification of the stylohyoid and stylomandibular ligament. Symptoms associated with Eagle Syndrome include orofacial and cervical pain, dysphagia, and pharyngeal foreign body sensation. Additionally, it can present with cerebrovascular symptoms due to the compression of adjacent neurovascular structures within the vicinity of the styloid process during rotation and extension of the neck. This report presents the case of a 33-year-old male with bilateral elongated styloid processes in whom the only symptom referred was recurrent syncope. The diagnosis was made years after the initial complaints and after several observations and imagings performed by different specialities. Surgical resection of the elongated process by the cervical approach was the treatment of choice. In patients with cerebrovascular symptoms, principally those induced by positional changes of the neck, Eagle syndrome should be considered in the differential diagnosis.

## Introduction

Eagle Syndrome (ES) is a symptomatic condition that results from the elongation of the styloid process (SP) of the temporal bone [1,2]. The first mention of elongated SP, along with its clinical and radiological findings, can be attributed to Watt Weems Eagle in 1937 [3]. Later, he described two distinct clinical conditions: classic stylohyoid syndrome and stylocarotid syndrome [4].

The first one (stylohyoid syndrome) occurs due to surgical trauma after tonsillectomy with symptoms like pain in the surgical area, sting pain, dysphagia, pain or discomfort while swallowing and sensation of a foreign body [2]. The scar resulting from tonsillectomy leads to contracture in the tonsil area, consequently creating tension on the elongated SP [1,5]. Furthermore, it might occur as a result of direct inflammation affecting the tissues and structures adjacent to the tip of the elongated SP, fracture of an ossified stylohyoid ligament caused by sudden head movement, followed by failure of healing and nonunion, and degenerative changes affecting the insertion of the stylohyoid ligament, resulting in the development of tendinosis at the insertion site [5]. In the second one (stylocarotid syndrome), complications within the central nervous system arise from the mechanical irritation caused by the elongated SP on the vascular walls of the internal and external carotid arteries [1,2].

In this report, we present a case of syncope related to neck rotation and extension associated with bilateral elongation of the SP.

## Case presentation

**Figure 1:**
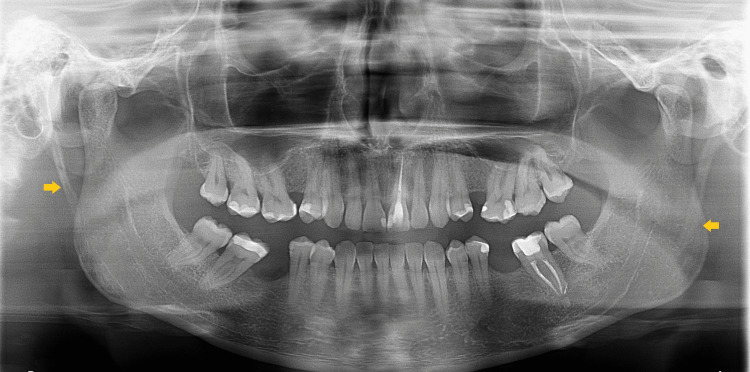
Panoramic radiograph showing a bilateral elongated styloid processes (yellow arrows).

A 33-year-old male was referred to our maxillofacial department with a five-year history of recurrent syncope. The episodes were preceded by prodromic symptoms that manifested when the patient turned his head to the left for a few seconds (like when shaving) and also with neck extension for a longer time (like drinking a bottle of water until it was finished). Spontaneous recovery occurred in less than a few seconds. Due to these recurrent syncope episodes, the patient made lifestyle changes in order to reduce head rotation and extension to prevent having syncopal episodes. The patient was previously followed by the family doctor and cardiology and neurology teams before being referred to our department. His dentist carried out a panoramic radiograph that showed bilateral elongated SPs (Figure 1).

**Figure 2:**
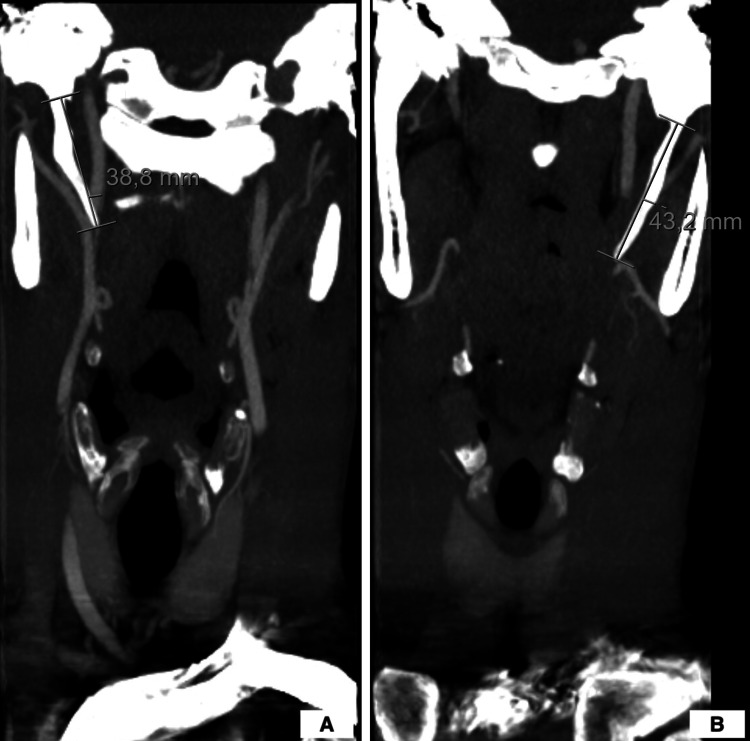
Bilateral styloid processes as measured by CT scan. (A) Right styloid process; (B) Left styloid process

The patient did not report any issues related to his neck area such as pain, discomfort, foreign body sensation, difficulty in swallowing or visual disturbances. The patient has no relevant medical/surgical history. During the physical examination, with the patient’s head held in an anatomical position, no distinctive sign or symptom was identified and neurologic examination were normal. Upon extension and rotation of the neck towards the left, the patient developed dizziness and weakness, which resolved within 10 seconds of repositioning the head back to its anatomical position. A systematic investigation was conducted. The computerized tomography (CT) of the neck was performed and demonstrated a bilateral elongated SP (38.8 mm on the right, 43.2 mm on the left) (Figure 2). 

**Figure 3:**
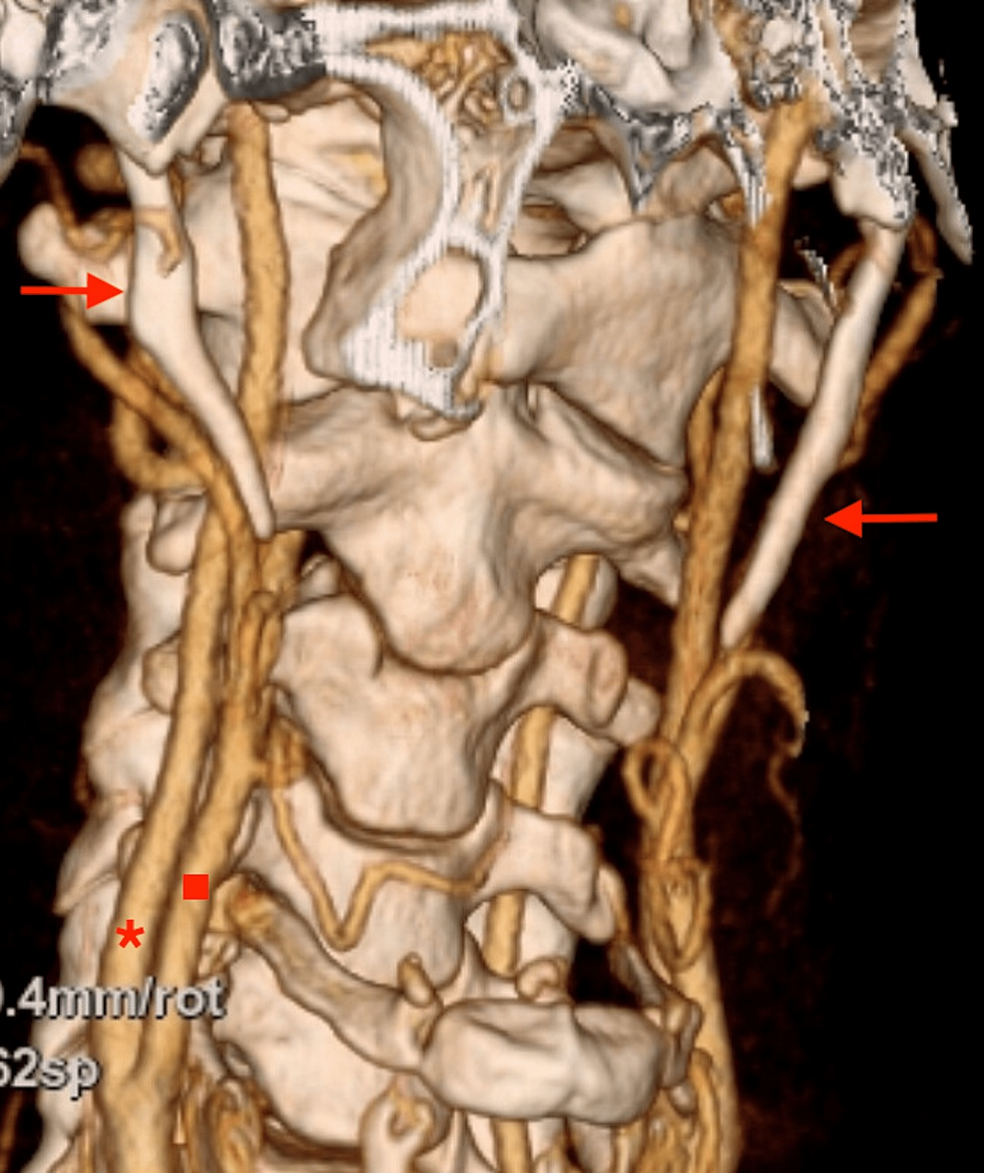
CT angiography (three-dimensional reconstruction) shows bilateral elongated styloid processes (indicated by two arrows) in relation to the external carotid artery (marked with a square) and the internal carotid artery (marked with an asterisk).

A CT angiography (CTA) of the patient's neck region was performed in the normal anatomical position and in all the positions that we previously observed to trigger the patient's symptoms of syncope; that is the neck extension and rotation to the left side (Figure 3). It confirmed a bilateral elongation of the SPs, both compressing the internal carotid artery respectively.

**Figure 4:**
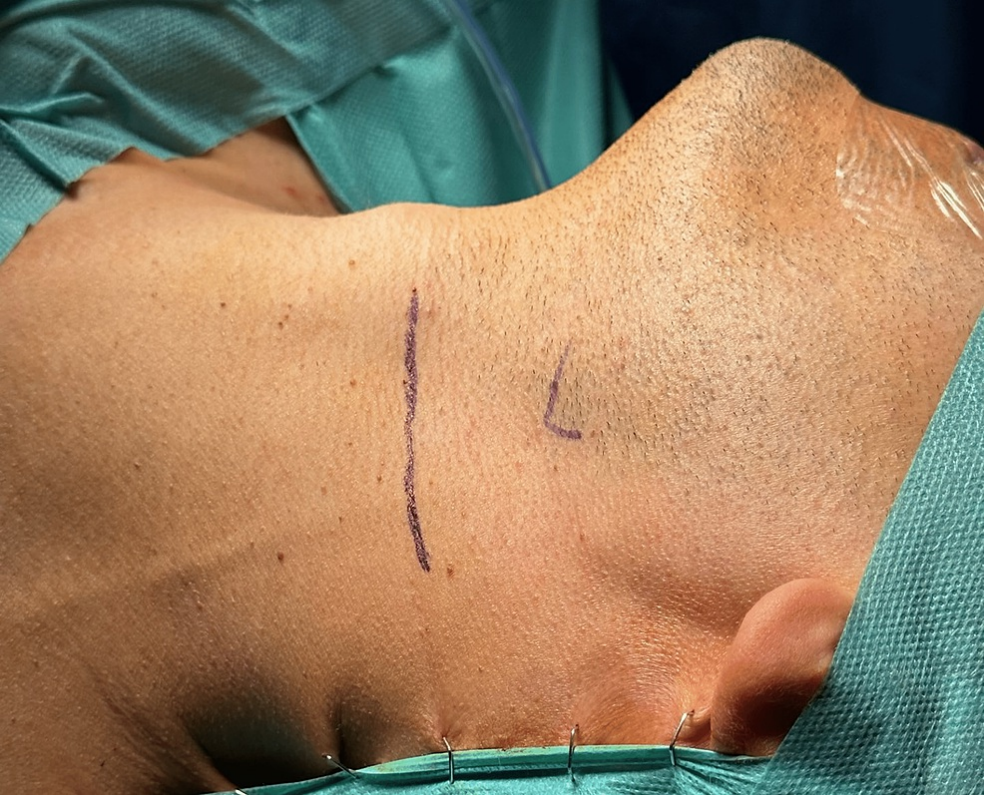
Cervical transcutaneous approach.

Due to the compression of the carotid arteries and subsequent stimulation of the sympathetic nerve plexus, syncope and other symptoms occurred and we concluded that surgical styloidectomy by cervical transcutaneous approach was the treatment of choice (Figure 4).

**Figure 5:**
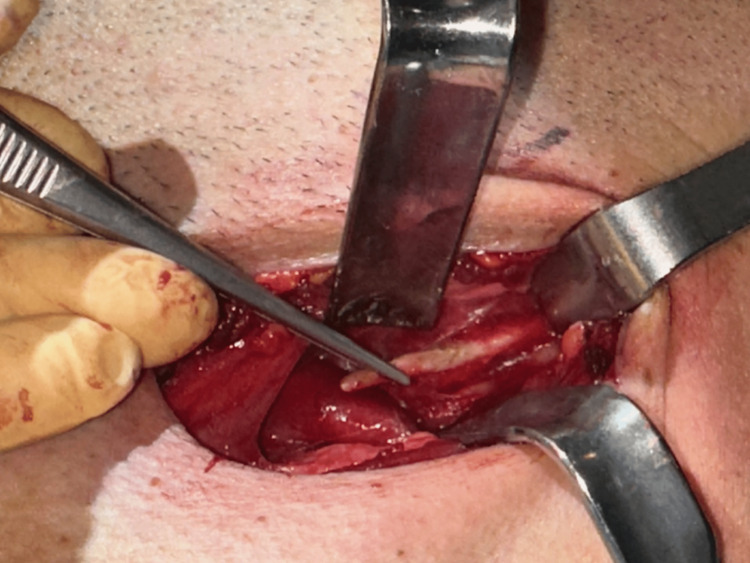
Intraoperative dissection.

**Figure 6:**
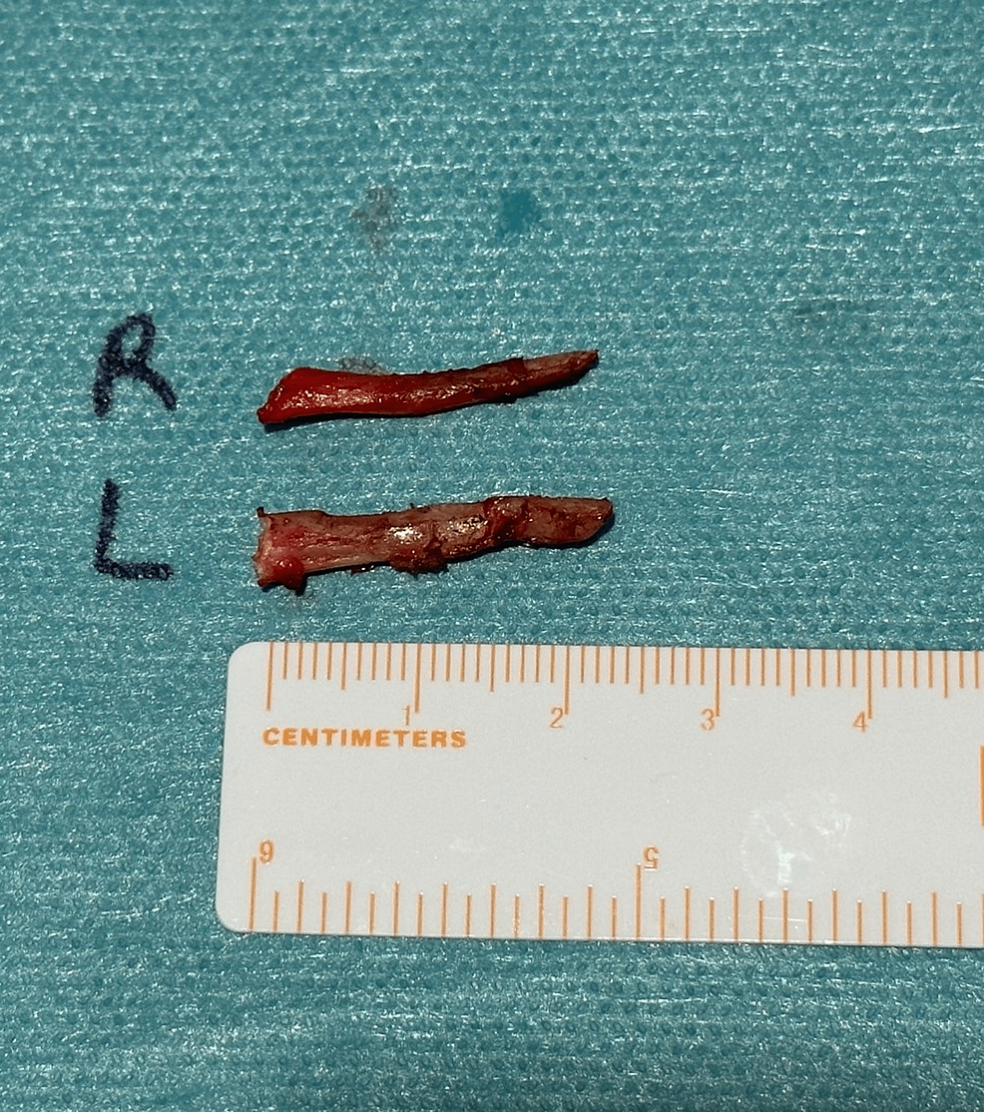
Styloidectomy specimen.

A skin incision was made 2cm below the inferior border of the mandible and the dissection was performed through the platysma muscle and the superficial layer of the deep cervical fascia. The submandibular gland was identified, followed by the retraction of the digastric muscle. Further dissection and palpation revealed the elongated SP. The SP was carefully cleaned to expose the bone, grasped near the skull base and fractured (Figures 5, 6).

**Figure 7:**
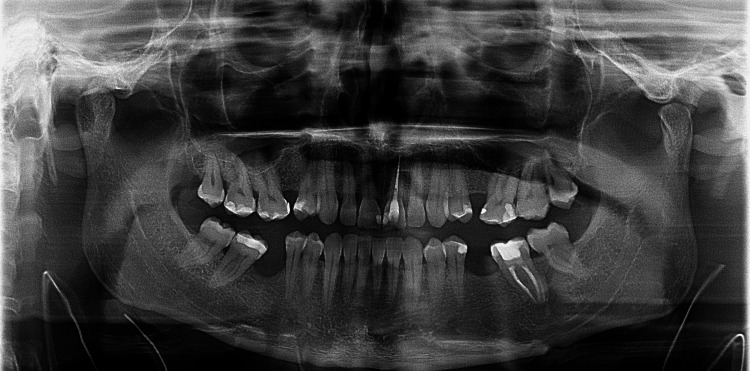
Postoperative panoramic radiograph.

Carotid artery exploration was not performed. Intraoperative and postoperative complications were not seen. A postoperative panoramic radiograph was also obtained (Figure 7). 

An implantable loop recorder, which was placed by the cardiology team at the onset of symptoms did not register any episode after surgery. The patient did report any further symptoms at the seven-month follow-up.

## Discussion

The SP is a cylindrical, thin projection of the temporal bone embryologically originating from the second pharyngeal arch. This is formed of one bone (SP), two ligaments (stylohyoid and stylomandibular), and three muscles (stylohyoid, stylopharyngeus, and styloglossus). The distal tip lies between the internal and external carotid arteries, oriented towards the medial and anterior directions. It is closely associated with significant vessels and nerves: the internal carotid and occipital arteries, the internal jugular vein and the glossopharyngeal, accessory, vagus and hypoglossal nerves. An unusually curved or elongated SP can potentially injure any of these structures [6].

A review of the literature by Badhey et al. in 2017 revealed that different authors defined different radiological lengths of the SP as the upper limit of normal (25 mm, 30 mm, and 40 mm) [1,7]. Although “normal SP length” is not constant in the literature, more than 30 mm is usually considered to be excessive [2,6,8]. Although a recent systematic review showed an estimated prevalence of 30.2% [9], the majority of these cases are asymptomatic with only 4% (approximately) presenting symptoms [2]. In the majority of cases, the elongation of the SP is bilateral. Nevertheless, while most symptomatic cases show elongation on both sides, the symptoms tend to manifest unilaterally [2]. Our case had a bilateral elongation of SP as mentioned in the literature, with symptoms that were correlated with the left side on prolonged cervical extension and rotation to the left.

The cerebrovascular symptoms of ES could be explained by several mechanisms: cerebral ischemia from vascular compression; injury to the carotid arteries with dissection or embolization; a vasovagal response elicited by mechanical stimulation of the carotid sinus through contact with the SP [10]. In cases of stylocarotid syndrome with a normal SP length, syncope can be attributed to the constriction of the internal or external carotid [2,11]. This could be accompanied by referred pain along the distribution of the artery, triggered by stimulation of the sympathetic nerve plexus associated with the artery [2,5,11]. Furthermore, although less common, symptoms may arise from direct compression on the carotid artery, including aphasia, visual disturbances, weakness, and even episodes of syncope [1,2,5,8]. Patients with this condition often report sudden onset and resolution of symptoms, which can be attributed to fluctuations in arterial flow [2]. In our patient, the rapid onset and subsequent resolution of symptoms, along with the lack of syncope when avoiding cervical hyperextension and left neck rotation movements are consistent with the vasovagal response.

Although panoramic radiographic imaging can be used for the diagnosis of ES, a CT scan provides us with a valuable anatomic relationship between SP and neighbouring neurovascular structures [1,2]. With CTA, the scanning time is reduced and it provides excellent visualization of both vascular structures and bone calcifications [2]. Doppler ultrasonography could be another option to measure arterial blood flow within the neck region with the patient's head in anatomical, extension, or rotation position in cases with suspected vascular compression. However, practical implementation of this approach is challenging as it becomes difficult to effectively scan the distal portion of the artery during rotation. Furthermore, the scanning process becomes even more challenging for patients to tolerate when vertigo or other complaints start [2].

The only effective treatment is a surgical shortening of the elongated SP [2,11]. Both intraoral and extraoral approaches are available [11]. The intraoral approach is a shorter procedure and does not result in scar tissue in the neck region [1,11]. Disadvantages include a limited field of view, the risk of deep neck infection after surgery, and uncontrolled bleeding [1]. The transcervical approach has the advantage of a wide surgical field and affords higher exposure to the carotid artery, while the formation of scar tissue on the skin is observed. It represents a surgical alternative that is both effective and safe, characterized by minimal associated morbidity [1,8]. In our perspective, patients with presumptive vascular involvement and stylocarotid symptoms are more carefully treated with the transcervical approach.

## Conclusions

ES, especially the stylocarotid form, should be considered in patients with neurological symptoms. To the best of our knowledge, this is the first case that has been reported with exclusive episodes of syncope related to ES. Considering that ES is underdiagnosed, it is important to consider the stylocarotid syndrome as a possible aetiology within its differential diagnoses, especially when sudden syncope results in a transitional change of the head position. A multidisciplinary approach is recommended when this diagnosis is presumable to exclude other causes of syncope. Panoramic radiography may be suggested as a preliminary investigation of SPs in such cases.

## References

[1] Bal KK, Ismi O, Esen K, Yilmaz IA, Vayisoglu Y (2018). A rare cause of recurrent cerebral ischemia and syncope: eagle syndrome. J Craniofac Surg.

[2] Demirtaş H, Kayan M, Koyuncuoğlu HR, Çelik AO, Kara M, Şengeze N (2016). Eagle syndrome causing vascular compression with cervical rotation: case report. Pol J Radiol.

[3] Eagle WW (1949). Symptomatic elongated styloid process; report of two cases of styloid process-carotid artery syndrome with operation. Arch Otolaryngol (1925).

[4] Eagle WW (1948). Elongated styloid process; further observations and a new syndrome. Arch Otolaryngol (1925).

[5] Chuang WC, Short JH, McKinney AM, Anker L, Knoll B, McKinney ZJ (2007). Reversible left hemispheric ischemia secondary to carotid compression in Eagle syndrome: surgical and CT angiographic correlation. AJNR Am J Neuroradiol.

[6] de Barros JF, Rodrigues MV, Barroso LA, Amado IC (2021). Eagle Syndrome: an underdiagnosed cause of orofacial pain. BMJ Case Rep.

[7] Badhey A, Jategaonkar A, Anglin Kovacs AJ (2017). Eagle syndrome: a comprehensive review. Clin Neurol Neurosurg.

[8] Esiobu PC, Yoo MJ, Kirkham EM, Zierler RE, Starnes BW, Sweet MP (2018). The role of vascular laboratory in the management of Eagle syndrome. J Vasc Surg Cases Innov Tech.

[9] Nogueira-Reis F, de Oliveira Reis L, Fontenele RC, Freitas DQ, Tabchoury CP (2022). Prevalence and features of elongated styloid process on imaging studies: a systematic review and meta-analysis. Clin Oral Investig.

[10] Todo T, Alexander M, Stokol C, Lyden P, Braunstein G, Gewertz B (2012). Eagle syndrome revisited: cerebrovascular complications. Ann Vasc Surg.

[11] David J, Lieb M, Rahimi SA (2014). Stylocarotid artery syndrome. J Vasc Surg.

